# Comprehensive genomic and transcriptomic analysis enables molecularly guided therapy options in peritoneal and pleural mesothelioma

**DOI:** 10.1016/j.esmoop.2025.104532

**Published:** 2025-04-01

**Authors:** L. Möhrmann, M. Werner, M. Oleś, L. Knol, J.S. Arnold, T. Mundt, N. Paramasivam, D. Richter, M. Fröhlich, B. Hutter, J. Hüllein, A. Jahn, C. Scheffold, E.E. Möhrmann, D. Hanf, S. Kreutzfeldt, C.E. Heilig, M.-V. Teleanu, D.B. Lipka, K. Beck, A. Baude-Müller, I. Jelas, D.T. Rieke, L.V. Klotz, R. Shah, T. Herold, M. Boerries, A.L. Illert, M. Allgäuer, A. Stenzinger, I.A. Kerle, P. Horak, C. Heining, E. Schröck, D. Hübschmann, S. Fröhling, H. Glimm

**Affiliations:** 1 Department of Translational Medical Oncology, National Center for Tumor Diseases (NCT), NCT/UCC Dresden, a partnership between DKFZ, Faculty of Medicine and University Hospital Carl Gustav Carus, TUD Dresden University of Technology, and Helmholtz-Zentrum Dresden-Rossendorf (HZDR), Dresden, Germany; 2 Translational Medical Oncology, Faculty of Medicine and University Hospital Carl Gustav Carus, TUD Dresden University of Technology, Dresden, Germany; 3 German Cancer Consortium (DKTK), Dresden, Germany; 4 Computational Health Informatics Program, Boston Children's Hospital, Harvard Medical School, Boston, USA; 5 Department of Internal Medicine I, University Hospital Carl Gustav Carus, TUD Dresden University of Technology, Dresden, Germany; 6 Computational Oncology Group (CO), Molecular Precision Oncology Program (MPOP), German Cancer Research Center (DKFZ), Heidelberg, Germany; 7 National Center for Tumor Diseases (NCT), NCT Heidelberg, a partnership between DKFZ and Heidelberg University Hospital, Heidelberg, Germany; 8 Institute for Clinical Genetics, University Hospital Carl Gustav Carus at TU Dresden, Dresden, Germany; 9 ERN GENTURIS, Hereditary Cancer Syndrome Center Dresden, Dresden, Germany; 10 Max Planck Institute of Molecular Cell Biology and Genetics, Dresden, Germany; 11 Division of Translational Medical Oncology, German Cancer Research Center (DKFZ), Heidelberg, Germany; 12 Section of Translational Cancer Epigenomics, Department of Translational Medical Oncology, German Cancer Research Center (DKFZ), Heidelberg, Germany; 13 German Cancer Consortium (DKTK), Heidelberg, Germany; 14 Molecular Precision Oncology Program, NCT Heidelberg and German Cancer Research Center (DKFZ), Heidelberg, Germany; 15 Charité Comprehensive Cancer Center, Universitätsmedizin Berlin, Freie Universität Berlin and Humboldt-Universität zu Berlin, Berlin, Germany; 16 Thoraxklinik, Heidelberg University Hospital, Heidelberg, Germany; 17 Department of Internal Medicine III, University Hospital, LMU Munich and Comprehensive Cancer Center, Munich, Germany; 18 Institute of Medical Bioinformatics and Systems Medicine, Medical Center—University of Freiburg, Faculty of Medicine, University of Freiburg, Freiburg im Breisgau, Germany; 19 Department of Internal Medicine I, Division of Hematology, Oncology and Stem Cell Transplantation, University Medical Center Freiburg, Freiburg im Breisgau, Germany; 20 Klinik und Poliklinik fur Innere Medizin III, Klinikum Rechts der Isar, School of Medicine, Technical University of Munich, Munich, Germany; 21 Institute of Pathology, University Hospital Heidelberg, Heidelberg, Germany; 22 Institute of Human Genetics, Heidelberg University, Heidelberg, Germany; 23 Innovation and Service Unit, German Cancer Research Center (DKFZ), Heidelberg, Germany; 24 Pattern Recognition and Digital Medicine Group, Heidelberg Institute for Stem cell Technology and Experimental Medicine (HI-STEM gGmbH), Heidelberg, Germany; 25 Translational Functional Cancer Genomics, German Cancer Research Center (DKFZ) Heidelberg, Heidelberg, Germany

**Keywords:** Key **w**ords: precision oncology, targeted therapy, pleural mesothelioma, peritoneal mesothelioma, homologous repair deficiency

## Abstract

**Introduction:**

Peritoneal, pericardial and pleural mesothelioma (PeM/PcM/PM) are rare and aggressive diseases with limited survival. Molecularly guided therapy is currently not part of standard care.

**Methods:**

This study integrates molecular and clinical data from 51 patients (among them 28 PM, one PcM, 21 PeM and one synchronous PeM/PM) enrolled in the National Center for Tumor Diseases and the German Cancer Consortium (NCT/DKTK) Molecularly Aided Stratification for Tumor Eradication Research (MASTER), a multicenter precision oncology registry trial addressing adults with rare advanced-stage cancers. Analysis comprised both somatic and germline whole exome sequencing/whole genome sequencing and transcriptome analysis leading to personalized treatment recommendations issued by a dedicated molecular tumor board. To assess clinical efficacy, progression-free survival (PFS) ratios comparing molecularly informed therapies (PFS2) to preceding systemic therapies (PFS1) were calculated. Efficacy of immune checkpoint inhibition applied during the observation period was assessed accordingly.

**Results:**

Cancer-related genes altered in more than 5 out of 44 assessable patients were *BAP1*, *CDKN2A*, *NF2*, *SETD2* and *TP53*. Somatic (*n* = 23) or germline (*n* = 9) alterations in homologous recombination-related genes were detected in 27/44 patients. In 21/44 cases, they were supported by positive combined homologous recombination deficiency scores or BRCAness signature. Following American College of Medical Genetics and Genomics guidelines, (likely) pathogenic germline variants in autosomal dominant cancer predisposition genes were found in 8/51 patients. Molecular tumor board recommendations were issued in 46 cases and applied in 6 cases. Mean PFS ratio was 2.45 (*n* = 5). Median PFS2 was 6.5 months (*n* = 6), median PFS1 was 4.0 months (*n* = 5). A total of 27 patients received immune checkpoint inhibition during the observation period leading to a mean PFS ratio of 1.69 (*n* = 19).

**Conclusions:**

In mesothelioma, comprehensive molecular analysis can provide valuable clinically actionable information. Molecularly informed therapy recommendations can lead to clinical benefit.

## Highlights

• WGS/WES and RNA sequencing-based comparative analysis of peritoneal and pleural mesothelioma.
• Tumor suppressor genes *BAP1*, *NF2*, *CDKN2A* and *PRBM1* are most frequently altered.
• Mutations indicating homologous repair deficiency are detected in 27/44 (61%).
• Immune checkpoint inhibition shows comparable efficacy in peritoneal and pleural mesothelioma.

## Introduction

Mesothelioma is a rare and aggressive tumor arising from the mesothelium, a thin layer of tissue lining the outer surface of all serous cavities including thorax, abdomen, pericardium and testes. Primary site is ∼80% pleural, ∼10% peritoneal and ∼10% pericardial or testicular.^1^, ^2^, ^3^ Pleural mesothelioma (PM) is known to be closely associated with asbestos exposure, with chronic inflammation being the main driver of carcinogenesis.^4^, ^5^, ^6^, ^7^ In addition, heterozygous germline alterations in *BAP1* leading to *BAP1* tumor predisposition syndrome can cause Mendelian distribution of mesothelioma occurrence across generations.^8^ Histologically, mesothelioma can be classified into epithelioid, biphasic or sarcomatoid differentiation.^9^ Epithelioid differentiation is associated with significantly longer overall survival (OS).^10^ Primary treatment of resectable PM includes cytoreductive surgery and perioperative systemic treatment with cisplatin or carboplatin, pemetrexed or, in case of unresectable tumors, chemotherapy or immune checkpoint inhibition (ICI) with ipilimumab and nivolumab.^11^^,^^12^ ICI in the aforementioned setting is approved by the Food and Drug Administration (FDA) and the European Medicines Agency (EMA). In peritoneal mesothelioma (PeM), treatment is heavily based on (cytoreductive) surgery followed by hyperthermic intraperitoneal chemotherapy. Systemic chemotherapy with cisplatin and pemetrexed is an option in neoadjuvant and adjuvant settings; ICI can be evaluated as an off-label treatment.^13^^,^^14^ Overall, prognosis is unfavorable with a median OS of <2 years depending on stage and histological subtype.^15^ The 5-year survival rate has been described as low as 2%.^16^ OS is significantly better in PeM than in PM.^17^

Studies investigating genomic alterations in both PM and PeM using mostly gene panel assays are available; some publications also include whole exome sequencing (WES), whole genome sequencing (WGS) or RNA sequencing.^18^, ^19^, ^20^, ^21^, ^22^, ^23^ However, there is a notable gap in systematic genome-wide studies of molecular alterations in PeM and the availability of clinical trials examining targeted treatment in molecularly stratified cohorts remains very limited. The MiST umbrella trial assessed molecularly guided treatments in BAP1/BRCA- and p16inkA4-deficient patients showing promising results but did not lead to changes in treatment recommendations for these subpopulations yet.^24^^,^^25^ Molecularly guided treatments are currently not standard practice in PM nor PeM.^11^^,^^13^ As treatment options become exceedingly scarce, especially in late-stage disease, further research exploring targeted therapy in mesothelioma is highly warranted.

The Molecularly Aided Stratification for Tumor Eradication Research (MASTER) program of the National Center for Tumor Diseases and the German Cancer Consortium (NCT/DKTK) aims at bridging this gap by whole-genome/exome and RNA sequencing as well as methylome analysis to enable a cross-institutional molecular tumor board (MTB) to provide evidence-based treatment recommendations.

Here, we use WES/WGS and RNA sequencing to describe the genomic landscape of both PM and PeM to detect limited but noticeable differences between entities and a surprisingly high number of homologous recombination (HR)-deficient patients overall. We identify a substantial number of targetable alterations and apply molecularly guided treatment within a clinically embedded setting.

## Methods

### NCT/DKTK MASTER

MASTER is a prospective precision oncology registry trial conducted by the NCT/DKTK addressing adults with rare advanced-stage cancer across histologies and providing a standardized workflow that leads to personalized therapy recommendations based on comprehensive molecular analysis.^26^^,^^27^ A semiweekly, multidisciplinary, multicenter MTB staffed with bioinformaticians, clinical geneticists, medical oncologists and pathologists was reviewing, interpreting and discussing results of WES/WGS, RNA sequencing and from June 2018 methylome analysis with access to medical records of presented patients. Based on publicly available knowledge at the time of each MTB meeting, clinical actionability of aberrations was determined, leading to molecularly informed therapy recommendations. Tumor board recommendations (TBRs) were prioritized by drug accessibility, assigned NCT molecular evidence level and expected efficacy based on existing (clinical) evidence.^28^ Therapeutic options improved over time. To enroll in MASTER, patients must have exhausted curative treatment options and be in good general condition (Eastern Cooperative Oncology Group performance status of 0 or 1).

### Ethics approval and consent to participate

Patients provided written informed consent for banking of tumor and control tissue, molecular analysis including germline analysis and the collection of clinical data under a protocol (S-206/2011) approved by the Ethics Committee of the Medical Faculty of Heidelberg University. The study was conducted in accordance with the Declaration of Helsinki.

### Clinical and statistical analysis

A total of 51 patients with PeM, pericardial mesothelioma (PcM) or PM diagnosed by their referring oncologists were included in the study cohort. All patients were enrolled between March 2014 and September 2024 in MASTER. Last follow-up was November 2024. Data were collected from five comprehensive cancer centers within the German Cancer Consortium (DKTK) network, namely Charité Comprehensive Cancer Center, Comprehensive Cancer Center Freiburg, Comprehensive Cancer Center München and the National Cancer Center Heidelberg and Dresden. Within DKTK, there are comprehensive cancer centers at 15 sites (Augsburg, Berlin, Cologne, Dresden, Essen/Düsseldorf, Erlangen, Frankfurt/Mainz, Freiburg, Heidelberg, Munich, Regensburg, Stuttgart, Tübingen, Ulm, Würzburg). Demographic data, ICD-O-3 morphology and topology, histopathological diagnosis, metadata regarding biopsy submission (dates, tumor cell content, locations), clinical information including full documentation of all oncologic diagnostic and therapeutic procedures carried out and MTB reports were obtained, assessed and captured in a central electronic data management system (ONKOSTAR) by medical curators based on prespecified guidelines for harmonized interpretation. MTB recommendations were based on WES/WGS and RNA sequencing. Clinical actionability of aberrations was determined based on publicly available knowledge at the time using databases such as PubMed, Clinvar, JKBjax or OnkoKB.^29^^,^^30^ Every tumor board report could include multiple therapy recommendations, each assigned a priority and an NCT/DKTK evidence level reflecting quality of underlying research.^28^ Recommended therapies were applied off-label or on-label at the discretion of the treating oncologist. Time between enrollment/diagnosis and death or last follow-up defined OS. Kaplan–Meier estimators were used to calculate median OS and progression-free survival (PFS), and log-rank tests were used to compare patient subgroups. Following Von Hoff et al.,^31^ we calculated PFS of recommended therapies (PFS2), of ICI (PFSi2) and each preceding systemic therapy (PFS1/PFSi1). To assess efficacy of recommended therapies or ICI we determined ratios between PFS2 and PFS1 (PFSr). Date of progression was determined based on radiologic findings or therapy change because of clinical deterioration, as assessed by the treating physician.

### Sample inclusion for molecular cohort analysis

All 51 patients underwent somatic and germline analysis within the MASTER workflow. To provide consistent and comparable results, we carried out secondary somatic (*n* = 44) and germline analyses (*n* = 51) retrospectively. Due to samples of low quality, seven patients did not undergo the secondary somatic and further downstream analyses. Three additional patients (Meso-20, Meso-30 and Meso-44) had to be excluded from copy number variation analysis due to either high level of degradation of the tumor sample, and thus unreliable segmentation, or incomplete workflow results due to computing system constrains. See Supplementary Data 1, available at https://doi.org/10.1016/j.esmoop.2025.104532 for more details.

### Genomic and transcriptomic analysis

Sample preparation and sequencing, technical details of WGS/WES and RNA-seq as well as downstream analyses are described in Supplementary Methods, available at https://doi.org/10.1016/j.esmoop.2025.104532.

### Germline analysis

Germline variant analysis was limited to 141 cancer predisposition genes and 43 genes were defined as HR-related (Supplementary Data 2, available at https://doi.org/10.1016/j.esmoop.2025.104532) as previously described.^32^^,^^33^ Further details are described in Supplementary Methods, available at https://doi.org/10.1016/j.esmoop.2025.104532.

### Availability of data and materials

Genome and RNA sequencing data generated in this study have been deposited in the European Genome-phenome Archive under the accession number EGAS00001007294. There is an institutional process in place to deal with requests for data transfer. GENCODE (release 19) was used for gene annotation and is publicly available. Raw clinical data are protected and are not available due to data privacy laws. Processed clinical data are available as Supplementary Data files, available at https://doi.org/10.1016/j.esmoop.2025.104532. The remaining data are available within the Article and Supplementary Data files, available at https://doi.org/10.1016/j.esmoop.2025.104532. Bioinformatics analyses were carried out using above-mentioned open-source software with parameters as described in each method section.

## Results

### Cohort

We enrolled 51 patients from the NCT/DKTK MASTER program. Primary site was in 21 cases (41%) peritoneal, in 28 cases (55%) pleural and in 1 case pericardial (2%). In the case of one patient (2%), pleural and peritoneal lesions were diagnosed synchronously. Epithelioid differentiation was identified in samples from 42 patients (82%), biphasic differentiation in samples from 5 patients (10%), sarcomatoid differentiation in 1 sample (2%) and diffuse dedifferentiation that could not be specified otherwise in samples from 2 patients (4%). In one patient, no data were available for differentiation (2%). Asbestos exposure was documented in 12 cases (24%), and smoking history in 13 cases (25%), with 7 patients both having been exposed to asbestos and having a smoking history. Median follow-up time was 8.7 months (range 0.03-49.9 months), 20 patients died during the observation period leading to a median OS from diagnosis of 41.3 months (range 2.5-90.9 months) and from enrollment of 12.3 months (range 0.03-49.9 months; see Supplementary Figure S1, available at https://doi.org/10.1016/j.esmoop.2025.104532). Cytoreductive surgery was carried out before enrollment in 25/28 patients (89%) with PM, 15/21 patients (71%) with PeM and the patient with synchronous PM/PeM. Nine patients (18%) had previously been diagnosed with another malignancy. Table 1 and Supplementary Data 3 and 4, available at https://doi.org/10.1016/j.esmoop.2025.104532, provide detailed cohort characteristics.

**Table 1:** Cohort description

Characteristics	No. of patients (%)
All	51 (100)
Sex	
Male	33 (65)
Female	18 (35)
Age, years	
18-29	3 (6)
30-39	4 (8)
40-49	10 (20)
50-59	14 (27)
60-76	20 (39)
Tissue molecular testing method	
WGS	42 (82)
WES	9 (18)
RNA	44 (86)
Germline analysis	51 (100)
Histologic diagnosis	
Epithelioid mesothelioma	42 (82)
Sarcomatoid mesothelioma	1 (2)
Biphasic	5 (10)
Diffuse malignant mesothelioma—not otherwise specified	2 (4)
No documentation	1 (2)
MTB recommendations	
Therapies recommended	46 (90)
Therapies applied	6 (12)
Number of systemic therapies before biopsy	
0	6 (12)
1	18 (35)
2	15 (29)
3	7 (14)
4 or more	4 (8)
No documentation	1 (2)
Immune checkpoint inhibition before biopsy	19 (37)
Location of primary site	
Pleura	28 (55)
Peritoneum	21 (41)
Synchronous (pleura and peritoneum)	1 (2)
Pericardium	1 (2)
Lymph node metastases at time of submission	
Present	25 (49)
Number of distant metastatic sites at time of submission	
0	27 (53)
1	8 (16)
2	11 (22)
3	5 (10)
Disease progression at time of diagnosis	
TNM stages (8th edition; pleural mesothelioma)	No. of patients
All	29 (100)
I	14 (48)
II	1 (3)
III	6 (21)
IV	8 (28)
Peritoneal Cancer Index^34^	Score out of 39
Mean (*n* = 6)	29.2

Cohort description including sex, age, tissue molecular testing method, number of germline analyses, histologic diagnoses, number of MTB recommendations and applications, number of systemic therapies before biopsy, locations of primary site, number of lymph node metastases at time of submission, number of distant metastatic sites at time of submission and information regarding disease progression at time of diagnosis.

MTB, molecular tumor board; TNM, tumor–node–metastasis; WES, whole exome sequencing; WGS, whole genome sequencing.

### Somatic alterations

**Figure 1:**
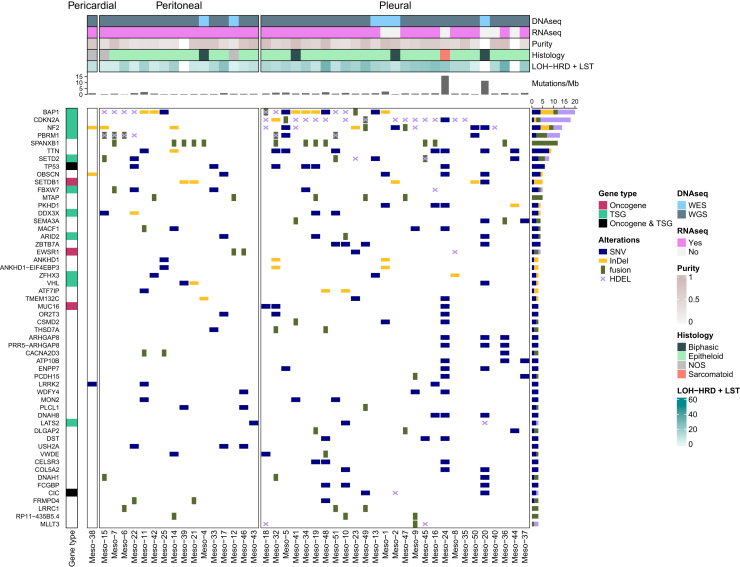
**Oncoplot showing sequencing information, measures of chromosomal instability and the most frequently mutated genes.** The 44 samples that were used for somatic analysis are represented in the columns. The top annotation bars include which analysis was carried out for which samples, the results of the histological assessment of each sample and measures of genomic instability. Overall genome ploidy is rounded to the nearest integer. The presence of SNVs, InDels, fusions and homozygous deletions (HDEL) are annotated in the body of the oncoplot by rectangles of different colors and sizes. Only genes with SNVs, InDels and/or fusions in three or more samples are shown here. The vertical bar indicates whether these genes are annotated as oncogenes and/or tumor suppressor genes (TSG) according to the Cosmic Gene Census (consulted October 2024). HRD, homologous recombination deficiency; InDel, insertion/deletion; LOH, loss of heterozygosity; LST, large-scale transitions; NOS, not otherwise specified; seq, sequencing; SNV, small nucleotide variant; WES, whole exome sequencing; WGS, whole genome sequencing.

WES (6/44; 14%) or WGS (38/44; 86%) was successfully carried out on tumor DNA and on DNA isolated from peripheral blood of one PcM, 16 PeM and 27 PM patients. RNA was successfully sequenced in 38/44 cases (86%). In PeM, we detected per sample a median of 23.5 non-silent small nucleotide variants (SNVs) ranging from 13 to 76, 2 small coding insertions/deletions (InDels) ranging from 0 to 7 and 11 fusions ranging from 0 to 17. In PM, we detected per sample a median of 36 non-silent SNVs ranging from 10 to 554, 4 small coding InDels ranging from 0 to 23 and 8 fusions ranging from 0 to 38. In one PcM sample, we observed a median of 42 non-silent SNVs, 4 InDels and 3 gene fusions. Patients Meso-20 and Meso-24, both PM, had hypermutated tumors (>5 non-silent mutations/megabase). Two patients, both PeM, were considered microsatellite instable (MSIsensor score >3.5). Cancer-related genes harboring SNVs, InDels or gene fusions in at least 15% of patients were in PeM *BAP1* and *FBXW7* (both *n* = 3), and in PM *BAP1* (*n* = 8), *NF2* (*n* = 6), *TP53* and *SETD2* (both *n* = 4). Figure 1 and Supplementary Figure S2, available at https://doi.org/10.1016/j.esmoop.2025.104532, present a detailed overview of somatic alterations by gene. Copy number analysis provided results in 41/44 cases (93%). On average, amplifications occurred on 11.3% and deletions on 19% of the covered genome. In both, PeM and PM, cancer-related gene *DROSHA* was amplified and *PRDM1*, *TNFAIP3* and *PBRM1* were deleted in >40% of samples. Additionally, deletion in *APOBEC3B*, *NF2*, *SMARCB1*, *CDKN2A*, *FBXW7*, *RB1* and *FANCA* was present in >40% of PM patients specifically, and in all those genes, besides *RB1*, deletion was significantly more abundant (*P* < 0.03, Fisher’s exact test) in PM than in PeM. Notably, homozygous deletion affecting *CDKN2A* also included *MTAP* in 10/14 (71%) patients. Regarding homozygous deletions, *CDKN2A* (*n* = 14), *BAP1* (*n* = 8) and *PBRM1* (*n* = 6) were affected most frequently. Homozygous deletions in *CDKN2A* occurred significantly more frequently in PM (PM 14/25; PeM 0/15; *P* = 0.0003, Fisher’s exact test). We detected BRCAness signature SBS3^35^ in 7/15 PeM (47%) and 18/26 PM (69%) and signature SBS4, associated with tobacco mutagens, in 3/15 PeM (20%) and 9/26 PM (35%) significantly with confidence intervals not including zero percent. Occurrence of signatures did not differ significantly between entities. Signatures are depicted in Supplementary Figure S3, available at https://doi.org/10.1016/j.esmoop.2025.104532. We observed overall limited differences between mutational landscapes of PeM and PM. Concerning tumor suppressor genes, however, mutations of *CDKN2A*/*MTAP* were detected exclusively in PM and mutations of B2M were absent in PeM.

### Germline alterations

Assessment of germline variants in 141 cancer predisposition genes among 51 patients revealed 13 heterozygous (likely) pathogenic variants (PGVs) in 13/51 patients (25%), 8 thereof in autosomal dominant cancer predisposition genes (3× *BAP1*, *ATM*, *BRCA2*, *CHEK2*, *NF1*, *SDHB*) and 10 in HR relevant genes (see Supplementary Data 5 and 6, available at https://doi.org/10.1016/j.esmoop.2025.104532). Among patients with PGVs, 6/13 had PeM (46%; PGVs in *ATM*, 2× *BAP1*, *CHEK2*, *FANCI*, *XPA*) and 7/13 had PM (54%; PGVs in *BAP1*, *BRCA2*, *FANCL*, *NBN*, *NF1*, *SDHB*, *WRN*). Mean age at diagnosis of patients with PGVs was similar to that of the other 38 patients. OS was similar between patients with and without PGVs (Supplementary Figure S4, available at https://doi.org/10.1016/j.esmoop.2025.104532). Among 9/51 patients with documented previous malignancies, 3/9 had a pathogenic germline variant (33%; *ATM*, *SDHB*, *WRN*). Meso-29, a patient with a likely pathogenic *BAP1* germline splice variant, had a family history of mesothelioma. Loss of heterozygosity (LOH) of the wild type allele occurred in 5/13 patients (38%) with PGVs in genes *ATM*, *BAP1*, *BRCA2*, *NBN* and *NF1*. LOH in *NF1*, associated with tumors in neurofibromatosis type I, had not yet been reported in mesothelioma. PGVs in HR relevant genes occurred in patients with documented asbestos exposure (5/10; Meso-16, *NBN*; Meso-19, *BAP1*; Meso-29, *BAP1*; Meso-33, *FANCL*; Meso-51, *WRN*). Taken together, we detect a high number of (likely) pathogenic germline variants that occur predominantly in HR relevant genes and mutually exclusive with LOH or somatic SNVs in *BAP1*.

### A high proportion of mesothelioma patients harbor tumors with homologous repair deficiency

To assess deficiency of HR, four molecular biomarkers were considered: somatic and germline alterations in HR-related genes, HR deficiency scores, calculated as the unweighted sum of LOH (HRD-LOH) and large-scale state transitions that are indicative of genomic instability and significant presence of mutational BRCAness signature. Mutations in HR-related genes were detected in 27/44 (61%) patients. Somatic mutations occurred in nine different genes in 23/44 (52%) patients. Germline mutations occurred in eight different genes in 9/44 (20%) patients. Within this subcohort, we detected positive HR deficiency scores or BRCAness signature individually in 5/27 (19%) patients, each. Both biomarkers were detected at the same time in 11/27 (41%) patients. Poly (ADP-ribose) polymerase (PARP) inhibition was recommended by the MTB in 23/46 patients (50%), but never applied.

### Molecularly informed treatment recommendations

**Figure 2:**
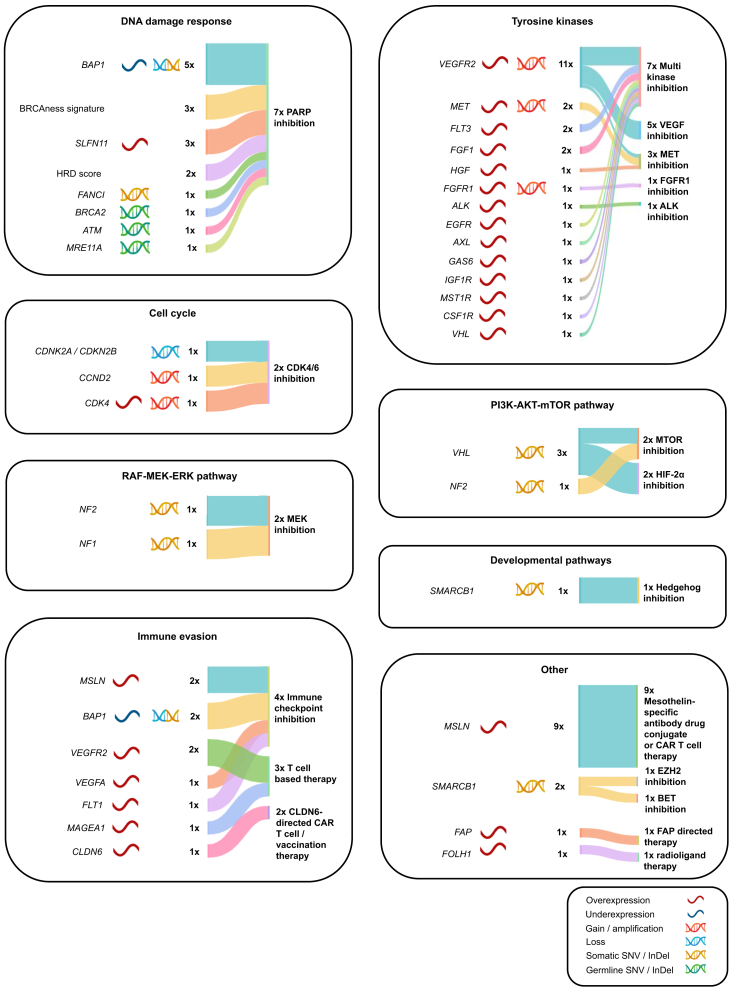
**Sankey plot depicting biomarkers and classes of derived therapy recommendations grouped by therapy baskets in peritoneal mesothelioma cases.** Sankey plot depicting biomarkers utilized by the MTB for patients with PeM, linked to the corresponding classes of derived therapy recommendations. Biomarkers are sorted by frequency and grouped into baskets as described by Horak et al., reflecting the pathways targeted by the recommended therapies^36^. For each basket group, the number of biomarkers, source of information (DNA or RNA), and type of alteration (expression, gain/amplification, loss/deletion, SNV, InDel) are indicated. In cases where biomarkers led to a recommendation involving combined therapy regimes associated with different therapy baskets (e.g. immune checkpoint inhibition and mTOR inhibition), biomarkers are depicted in both relevant baskets and linked to each corresponding treatment class. InDel, insertion/deletion; MTB, molecular tumor board; PeM, peritoneal mesothelioma; SNV, small nucleotide variant.

**Figure 3:**
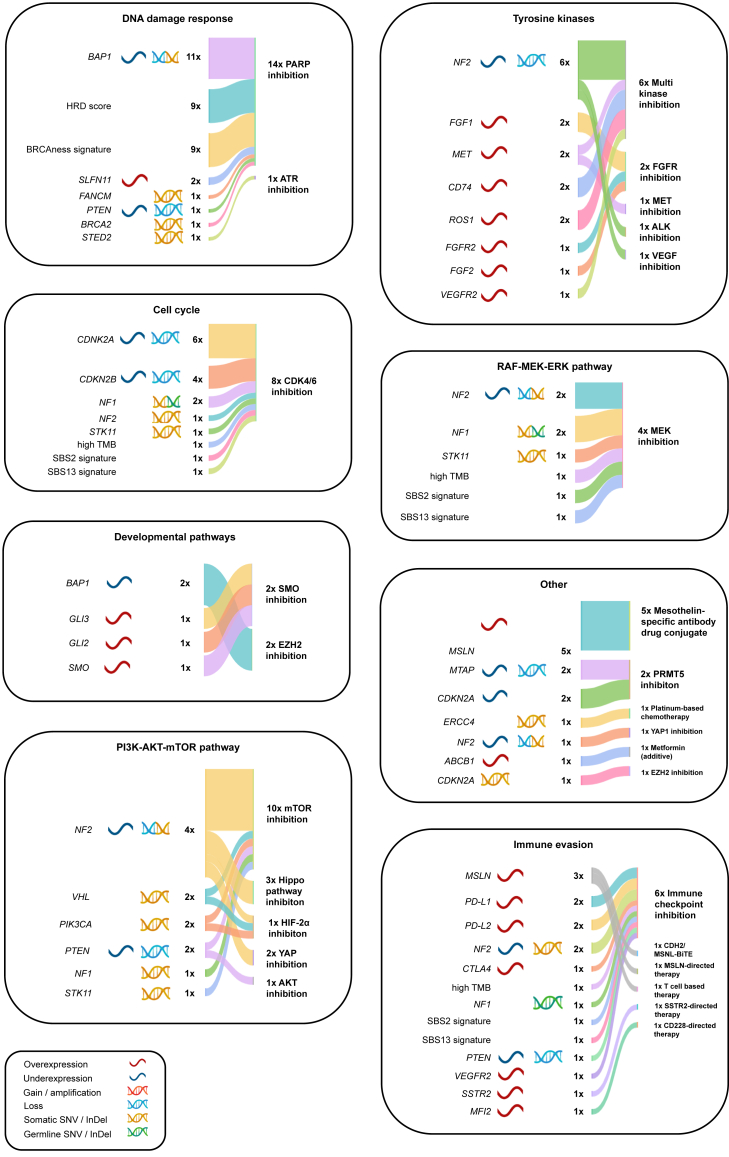
**Sankey plot depiciting biomarkers and classes of derived therapy recommendations grouped by therapy baskets in pleural mesothelioma cases.** Sankey plot depicting biomarkers utilized by the MTB for patients with PM, linked to classes of derived therapy recommendations. Biomarkers are sorted as described in Figure 2.^36^

Some 46/51 patients (90%) received 130 therapy recommendations issued by a dedicated MTB (see Supplementary Data 7, available at https://doi.org/10.1016/j.esmoop.2025.104532). In 3/51 cases (6%), patients died before the MTB could convene. In 2/51 cases (4%), tumor cell content was insufficient for analysis. Patients received a median of three recommendations (range 1-6). Median turnaround time between sample submission and TBR was 2.4 months ranging from 1.2 to 5.4 months. An NCT molecular evidence level was assigned to each recommendation as previously described.^28^ These were: m1a/b/c: 20/130 (15%; evidence in the same entity), m2a/b/c: 64/130 (49%; evidence in another entity), m3: 31/130 (24%; preclinical evidence), m4: 15/130 (12%; scientific rationale). The most often recommended treatment class was tyrosine kinase inhibition (33/130). Respective TBRs were equally distributed between PeM (19/33 TBRs) and PM (13/33 TBRs), as were TBRs for PARP inhibition (23/130; PM: 14/23 TBR; PeM: 8/23 TBR). Mammalian target of rapamycin (mTOR) inhibition was recommended mainly in PM (17/23 TBRs) and CDK4/6 inhibition was recommended almost exclusively in PM (8/10 TBRs), predominantly based on CDKN2A/B alterations (6/8 TBRs). All used biomarkers and derived TBRs are depicted in Figures 2 and 3.

### Clinical outcome of applied molecularly informed therapies

**Figure 4:**
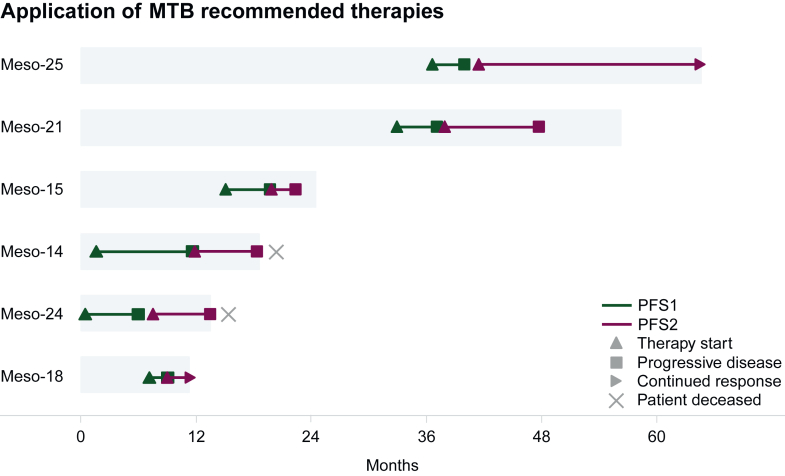
**Swimmer plot depicting application of molecular tumor board (MTB) recommended therapies.** Every bar depicts the duration of disease from diagnosis to end of follow-up or in case of bars annotated with x death. Plotted inside the bars is progression-free survival of the first applied molecularly guided therapy (PFS2, red) and its last preceding systemic therapy (PFS1, green). Continued response beyond end of observation period is marked with an arrow.

Recommended therapies were applied in 6/51 patients (12%), 40/51 patients (78%) received TBRs without documented application. In 25/40 cases (63%), this was due to a short follow-up period, with no documentation available on therapies administered beyond the MTB date. In 8/40 cases (20%), patients received an alternative therapy, and in 7/40 cases (18%), patients died without documentation of a systemic therapy between timepoint of tumor board and death. Three patients received ICI. mTOR inhibition, VEGFR inhibition, and peptide receptor radionuclide therapy were applied in one patient, each. To assess clinical benefit, we calculated the ratio (PFSr) between PFS of MTB recommended therapies (PFS2) and that of each preceding systemic therapy (PFS1), as described in previous large-scale precision oncology trials.^27^^,^^37^^,^^38^ Overall, PFS1 ranged between 2.0 and 5.5 months with a median of 4.0 months (*n* = 5). In Meso-22, PFS1 could not be determined because systemic therapy was administered as first line in an adjuvant setting. PFS2 ranged between 2.4 and 23.0 months with a median of 6.5 months (*n* = 6; Supplementary Figure S5A and B, available at https://doi.org/10.1016/j.esmoop.2025.104532). Mean PFSr was 2.45 (range 0.5-7.1; *n* = 5), PFSr was >1.3 in 2/5 evaluable cases. Figure 4 depicts PFS2 and PFS1 in the context of patient survival. One patient received a second TBR after PFS2. Best response during recommended treatment (*n* = 7) was partial response (PR) in two applications, stable disease (SD) in four applications and progressive disease (PD) in one case. This translates into an overall response rate (ORR) of 29% and a disease control rate (DCR) of 86%. There was a trend towards prolonged OS from enrollment for patients who received recommended therapies (*n* = 6; median not reached) compared with those who did not (*n* = 45; median 11.2 months; log-rank test; *P* = 0.113). The two patients with PFSr >1.3 responded exceptionally well to applied TBRs: Meso-25 was treated with a combination of nivolumab and ipilimumab based on *BAP1* LOH, several focal deletions and an A203G SNV entailing a PFS2 of 23.0 months until end of follow-up and a PFSr of 7.1. Meso-21 initially received mTOR inhibition with everolimus entailing a PFS2 of 9.7 months and a PFSr of 2.4. After PFS2, the HIF-2α inhibitor belzutifan was applied, leading to a PFS3 of 8.0 months. No further progression was observed until end of follow-up. Both recommendations were based on a p.75_76del deletion and LOH in *VHL.* Three patients did not achieve PFSr >1.3: Meso-15 was treated with nivolumab and ipilimumab based on loss of BAP1 and progressed after 2.6 months. Meso-18 underwent SSTR2-directed peptide receptor radionuclide therapy based on SSTR2 RNA overexpression and was lost to follow-up due to moving to another country. Meso-24 was treated with pembrolizumab and gemcitabine following the detection of a high mutational burden and an *NF2* frameshift alteration and achieved a PFS2 of 5.9 months after a first-line therapy with carboplatin/pemetrexed that lead to a PFS1 of 6.1 months.

### Immune checkpoint inhibition during observation period

The MTB identified biomarkers predicting efficacy of ICI in 9/46 cases (20%), most frequently *KDR* (*n* = 3), *NF2* (*n* = 2) and *BAP1* (*n* = 2). In total, 27 patients received ICI during the observation period (see Supplementary Data 9, available at https://doi.org/10.1016/j.esmoop.2025.104532). Some 3/27 patients (11%) received ICI based on TBRs, 24/27 patients (89%) based on physicians’ choice. A total of 19/28 PM patients (68%) received ICI: nivolumab/ipilimumab in 8 cases, nivolumab in 6 cases and pembrolizumab in 5 cases. Some 7/21 PeM patients (33%) received ICI: nivolumab/ipilimumab in 4 cases, nivolumab in 1 case and pembrolizumab in 2 cases. One patient with PcM received nivolumab/ipilimumab. To assess clinical benefit, we calculated the ratio (PFSr) between PFS of the first applied ICI (PFS2) and PFS of the preceding systemic therapy (PFS1). Mean PFSr was 1.69 (*n* = 19), PFSr was >1.3 in 7/19 assessable cases (37%). Mean PFSr in PM was 1.49 (*n* = 13), mean PFSr in PeM was 2.51 (*n* = 5). PFSr of the patient with PcM was 0.17. Mean PFSr during application of ipilimumab/nivolumab was 1.36 (*n* = 11), during application of pembrolizumab 2.39 (*n* = 5) and during application of nivolumab 1.72 (*n* = 3). Median PFS1 was 5.5 months (range 1.1-18.8 months; *n* = 19) and median PFS2 was 4.5 months (range 1.4-23.0 months; *n* = 26; Supplementary Figure S5C and D, available at https://doi.org/10.1016/j.esmoop.2025.104532). In seven cases, PFS1 could not be determined as ICI was administered in the first line. Best response during first ICI was PR in 7/26 cases, SD in 6/26 cases and PD in 13/26 cases. This translates into an ORR of 27% and a DCR of 50% (*n* = 26). In Meso-32, PFS2 could not be determined because ICI was applied as neoadjuvant therapy.

## Discussion

PM and PeM presented with predominantly inactivating mutations in well-known tumor suppressor genes *BAP1*, *NF2*, *CDKN2A* and *PRBM1*. *SETDB1* was the only oncogene that was altered in more than three patients. This is in line with previous findings in PM.^18^^,^^21^ There have been recent advances in addressing these alterations therapeutically. For example, the YAP inhibitor IAG933 induced deep tumor regression in NF2-altered mesothelioma models.^39^ A phase I clinical trial testing IAG933 is currently underway (NCT04857372). Furthermore, studies using TP53 reactivators are ongoing, but specific data regarding mesotheliomas are still missing.^40^ *CDKN2A* and *MTAP* were mainly inactivated by homozygous deletions and only affected in PM, although this finding may be due to low patient numbers overall. Structural variants and fusions occurred frequently in both PeM and PM underscoring the value of in-depth molecular analysis beyond panel-based NGS.

We evaluated germline alterations uncovering a high PGV frequency of 16% in autosomal dominant cancer predisposition genes, which is concordant with previous reports of PGVs being more prevalent in mesothelioma than in other types of adult cancer.^32^^,^^41^, ^42^, ^43^, ^44^ Observed PGVs occurred predominantly in genes related to HR and mutually exclusive with LOH in *BAP1* indicating a functional impact of impaired homologous repair on tumor emergence. Additionally, both patients with a PGV in *BAP1* had been exposed to asbestos fiber, which further implies mutually reinforcing genetic and environmental risk factors.^45^

Previous studies in mesothelioma suggest limited efficacy of PARP inhibition without patient stratification.^24^^,^^46^ A portion of patients, however, do benefit. No predictive biomarkers could yet be identified with *BAP1* and *BRCA1* mutation status being investigated unsuccessfully. To improve stratification efforts, we assessed the proportion of patients potentially benefiting from PARP inhibition through a comprehensive evaluation of HR-deficiency biomarkers, incorporating somatic and germline alterations in HR relevant genes, HRD scores and BRCAness signature SBS3,^35^ all of which have been associated with efficacy of PARP inhibition in other tumor entities before. This led to detection of impaired HR in a substantial number of patients. We hypothesize that co-occurrence of several HR biomarkers in an individual patient may lead to higher susceptibility to PARP inhibition. We propose to primarily consider this substance class when multiples of the aforementioned HR biomarkers are positive. Our data show that a relevant proportion of mesotheliomas fulfill these criteria indicating that further stratified clinical research has the potential to benefit a substantial number of patients.

Using in-depth genomic and transcriptomic analysis, we identify a diverse set of targetable alterations: 130 issued TBRs address 79 unique actionable biomarkers. Of those recommendations, 65% were based on available clinical data at the time rather than preclinical research, underscoring the quality of available biomarkers. Tyrosine kinase inhibition was being primarily proposed in PeM (58% of TBRs) and mTOR inhibition and CDK4/6 inhibition more frequently proposed in PM (74%/80% of TBRs). A differentiated perspective of targeted treatment in PeM and PM may be beneficial.

Recommended drugs were applied in 6/51 patients (12%). This rate is lower than the rates reported in similarly structured studies (16%-35%).^27^^,^^37^^,^^38^ The low number of therapy applications presents a relevant limitation to the generalizability of our results, which warrants further research to improve robustness. It can be partially attributed to an extended enrollment period until manuscript submission. This approach aimed to maximize collection of molecular data on this very rare entity. Consequently, in many cases, there was insufficient time for adequate follow-up to detect therapy applications. As predominantly patients with tumors of advanced stage and histology are enrolled in MASTER, progressions between enrollment and application of a given TBR occur regularly. The median turnaround time from sample submission to TBR was 2.4 months, in line with prior studies.^47^ Even though we see this as acceptable given the complexity of necessary processes, we acknowledge that there is a need for further improvement. However, time spans between tumor board and application can be critically long as access to recommended drugs is difficult. Targeted trials are rarely available for mesothelioma patients and regular cost coverage necessitates lengthy applications with health insurance providers.

Molecularly informed therapies lead to substantial clinical efficacy with a mean PFSr of 2.45 and a DCR of 86% in already pretreated patients. Meso-25 experienced response for 23 months whilst remaining without progression until the end of follow-up. Meso-21 responded for 9.7 months and could then again benefit from another therapy recommendation for 8.0 months without progression until the end of follow-up. Both cases highlight the potential benefit of molecularly guided treatment in mesothelioma. Transferability of these findings is limited by number of patients as PMs and especially PeMs are very rare tumors. Still, these results showcase promising targets for subsequent research.

Systemic therapy currently relies on chemotherapeutic regimes and ICI.^11^^,^^48^ For PM, combination of anti-programmed cell death protein 1 (PD-1) antibody nivolumab and anti-cytotoxic T-lymphocyte associated protein 4 (CTLA-4) antibody ipilimumab is FDA/EMA-approved whereas clinical evidence indicating efficacy of ICI in PeM is still limited.^14^ We observed frequent application of pembrolizumab and nivolumab both in PM and PeM showcasing clinical efficacy across entities and substances.

As there are very few molecularly stratified trials and published results could so far not identify any predictive biomarkers in mesothelioma,^24^^,^^25^^,^^46^ further efforts to integrate multi-omics and clinical data are highly warranted. Prospective precision oncology registry trials such as MASTER bridge this gap by evaluating a broad variety of molecular biomarkers in a clinically embedded setting. Especially RNA sequencing is necessary to detect clinically relevant overexpression of primarily tyrosine kinases and addressable antigens. Patients do not need to visit a study center and referring oncologists apply therapy recommendations regularly on site within their practice, thus enabling molecularly informed treatment even in remote regions. This approach provides invaluable real-world data with high transferability to similarly structured environments.

Conclusion

We provide relevant insight not only into underlying biologic mechanisms, but also therapeutic potential using multi-omics analysis. Differences in genomic alterations between entities are noticeable but limited. Composite biomarker analysis leads to identification of a relevant subgroup of HR-deficient patients. ICI leads to similar response in PM and PeM. Finally, molecularly informed therapy can provide substantial clinical benefit for patients with peritoneal and pleural mesothelioma.
