# Supplementary material for: Comprehensive genomic and transcriptomic analysis enables molecularly guided therapy options in peritoneal and pleural mesothelioma
Source: ESMO Open. 2025 Apr 1;10(4):104532. doi: 10.1016/j.esmoop.2025.104532 (PMC11999262; doi:10.1016/j.esmoop.2025.104532)
Supplement: Supplementary Methods [file mmc1.docx]

Supplementary Methods

**Sample preparation and sequencing**

Nucleic acid was extracted from solid tissue samples using Allprep DNA/RNA/miRNA Universal Kit (Qiagen) or QIAamp DNA mini (Qiagen), from formalin-fixed paraffin-embedded tissue samples using GeneRead DNA FFPE Kit (Qiagen) and for circulating blood using QIAamp DNA Blood Mini (Qiagen) or QIASymphony DSP DNA Mini Kit (Qiagen). Quality control and quantification was performed with Qubit 2.0 Fluorometer (Invitrogen) and a TapeStation 2200 system (Agilent).

Libraries were produced using Illumina TruSeq Nano (100ng input) for the whole genome sequencing and with Agilent SureSelect All Exon Kit v5 or v5 + UTRs (200ng input) for the whole exome sequencing, and sequenced using Illumina HiSeq 2500/3000/4000/X Ten, Illumina NovaSeq 600 or Illumina NovaSeq X Plus. Samples were processed for the Molecular Precision Oncology Program of the NCT Heidelberg by facilities at DKFZ. Those included Sample Processing Laboratory, Genomics and Proteomics Core Facility and Omics IT and Data Management Core Facility.

**Nucleotide sequence alignment**

Reads obtained with sequencing of DNA were mapped to the reference consisting of human genome (1000 Genomes Phase 2 of the Genome Reference Consortium; version hs37d5) and a genome of Enterobacteria phage phiX174, using BWA mem (version 0.7.15). Default settings were modified by setting parameter -T to 0, resulting in reporting all alignments in the output. BAM files were sorted using bamsort (biobambam package, version 0.0.148), and duplicates were marked with markdup (Sambamba package, version 0.6.5) ^1^.

**Calling of somatic single nucleotide variants, small insertions and deletions and structural variation**

Somatic calls of single nucleotide variants, small insertions and deletions as well as structural variants were done for each tumor/control sample pair.

SNVs were detected using in-house pipeline consisting of SAMtools mpileup and BCFtools ^2^ with filtering steps as described previously ^3–6^. In particular, the calling was made on reads with the minimum mapping quality of 30 (-q 30 in mpileup). SNVs with at least one high-quality non-reference base at a given position (-vcgN -p 2.0 in BCFtools) were preliminarily selected. The summary of observed bases in a control sample for each SNV position was gathered using mpileup. SNVs were then further annotated with ANNOVAR (version November 2014) using GENCODE (release 19). Downstream filtering was based on factors such as low support of the alternative allele, PCR strand bias (WGS-specific), sequencing strand bias, significant bias in the PV4 field of the mpileup output and those located in tandem repeats and other read-attracting regions. Calls with low confidence score were discarded. Non-silent SNVs are those annotated as stopgain, stoploss, missense or splicing (intronic region within two base pairs from an exon boundary).

InDels were detected using Platypus (version 0.8.1). Calls flagged as PASS and those which remained after custom filtering were annotated with ANNOVAR (version February 2016). InDels located within a coding sequence and at splice sites were selected for the analysis.

SVs were detected using Sophia (<https://bitbucket.org/utoprak/sophia/src/master>). Only the variants with a confidence score of 5 were considered.

**Detection of copy number variants**

Copy number variants (CNVs) were detected using ACEseq (v5.1.0) ^7^ for WGS or CNVkit (v. 0.9.3) ^8^ for WES. Ploidy and tumor cell content were derived based on segments with at least 20 heterozygous SNPs using a method implemented in ACEseq. In short, the algorithm tests each possible combination of ploidy (range 1.0–6.5) and tumor cell content (range 0.15-1.0) to find the local minima, and returns them as possible optimal solutions. If more than one optimal solution was suggested, one was chosen in the course of visual evaluation. Segments with copy number above 0.7 and below 0.7 from the tumor sample overall ploidy were annotated as amplifications and deletions, respectively. CNV results are missing for three samples. Degradation of tumor sample of Meso-20 lead to over-segmentation of CNV profile. In case of Meso-39 and Meso-44, CNV workflow failed to complete successfully, requiring manual intervention to proceed. Therefore, those samples were excluded due to system constraints and the need for reproducibility. Loss of heterozygosity (LOH-HRD) and large-scale state transitions (LST) scores, sum of which was used for determination of chromosomal instability, were obtained with respective CNV calling software.

**Determination of microsatellite instability**

MSIsensor-pro (version 1.2.0) ^9^ was used for determination of the microsatellite instability status in patients whose both control and tumor samples had a minimum sequencing coverage of 15 for exomes and 20 for genomes. 1000 genomes reference was used to build the list of homopolymers and microsatellites of 33,386,244 loci in total.

**Mutational Burden**

The mutational burden was defined as the sum of non-silent SNVs and InDels per megabase of genome (for WGS) or exome (for WES). For WES, only mutations within the range of the library probes were considered.

**RNA sequencing and gene fusion detection**

Libraries were prepared using Illumina TruSeq RNA (with 1000ng RNA), and since February 2016, using Illumina TruSeq mRNA stranded protocol (with 500ng total RNA). They were sequenced using Illumina HiSeq 4000, HiSeq X Ten, NovaSeq 6000 or NovaSeq X Plus. STAR 2.5.1b ^10^ was used for mapping of the sequencing reads to the reference genome defined earlier in the section describing DNA sequencing. Gene fusions were obtained using Arriba (version 2.4.0) ^11^ and only those called with high confidence were further analyzed.

**Mutational signatures**

Mutational signatures were extracted from all SNVs identified in tumor samples (for WES, the SNVs within the range of library target probes) using R/Bioconductor package YAPSA (version 1.14) ^12^ and Cosmic single base substitution signatures (version 2) ^13^. Two samples with 50 SNVs or less were excluded from the analysis. For WES samples, mutational catalogue was corrected with factors corresponding to the appropriate target capture which was used for library preparation.

Subsequently the mutational catalogue was scaled to the size of the genome. Mutational signatures were extracted and confidence intervals were calculated per sample using signature specific absolute cutoffs with cost factor 6. Signatures were considered significant if the confidence interval of the signature for that sample did not include zero.

**Technical details of germline analysis**

Retrospective germline SNV and InDel calling in both control and tumor samples using a platypus-based in-house workflow was performed ^14^. Rare variants were annotated with VEP (version 104) and additional VEP plugins (dbNSFP, CADD, GeneSplicer, dbscSNV). Somatic variants incorrectly assigned as germline were detected using TiNDA and excluded (44). Rare germline variants were subsequently manually inspected and annotated according to ACMG/AMP guidelines ^15^. Gene-specific recommendations were applied when appropriate ^16^. For evaluation of biallelic inactivation and contribution of germline variants to tumor development, retrospective loss-of-heterozygosity annotation of the wild type allele was performed with CNVkit (v. 0.9.3) for WES and ACEseq (v. 5.1.0) for WGS. Genetic counseling was recommended for all patients with a diagnosis of a cancer predisposition syndrome.

**HR related candidate genes and selected references**

(Gene - PubMed Identifier)

ATM 26510020, 24841718

ATR 25965342, 27708213

BAP1 22683710, 27447864, 28389374, 24347639

BARD1 27197561, 32444418

BLM 28912125, 32444418

BRCA1 26510020

BRCA2 26510020

BRIP1 24240112, 26709662

CDK12 24240700

CHEK1 16912188

CHEK2 26510020

ERCC3 26510020

ERCC4 26074087

FAM175A 32444418

FANCA 26510020, 23325218, 15650050

FANCB 23325218, 15650050

FANCC 25609062, 12093742

FANCD2 27264184

FANCE 12093742

FANCF 23325218, 15650050

FANCI 23325218, 15650050

FANCL 23325218, 15650050, 17352736

FANCM 23325218, 15650050

HDAC2 26510020

MLH3 26510020

MRE11 26510020

NBN 26510020

PALB2 26510020

PTEN 20049735, 20944090, 21468130, 23239809, 24625059

RAD18 25417706, 26056084

RAD50 32444418, 29709199

RAD51 26510020

RAD51B 23239809, 24278037, 29465803

RAD51D 21822267**,** 22652533

RAD54L 16912188, 26056084, 26669450, 28223274

RECQL4 29229926, 27320928

RPA1 16912188, 23239809

SETD2 24931610, 30818762

SLX4 22354996

UBE2T 30715513

XRCC3 174795, 23512992, 23760496, 25028150, 29465803

XRCC9 12861027

WRN 12934712

References

1. Tarasov A, Vilella AJ, Cuppen E, Nijman IJ, Prins P. Sambamba: fast processing of NGS alignment formats. *Bioinforma Oxf Engl*. 2015;31(12):2032-2034. doi:10.1093/bioinformatics/btv098

2. Danecek P, Bonfield JK, Liddle J, et al. Twelve years of SAMtools and BCFtools. *GigaScience*. 2021;10(2):giab008. doi:10.1093/gigascience/giab008

3. Jones DTW, Jäger N, Kool M, et al. Dissecting the genomic complexity underlying medulloblastoma. *Nature*. 2012;488(7409):100-105. doi:10.1038/nature11284

4. Jones DTW, Hutter B, Jäger N, et al. Recurrent somatic alterations of FGFR1 and NTRK2 in pilocytic astrocytoma. *Nat Genet*. 2013;45(8):927-932. doi:10.1038/ng.2682

5. Reisinger E, Genthner L, Kerssemakers J, et al. OTP: An automatized system for managing and processing NGS data. *J Biotechnol*. 2017;261:53-62. doi:10.1016/j.jbiotec.2017.08.006

6. Möhrmann L, Werner M, Oleś M, et al. Comprehensive genomic and epigenomic analysis in cancer of unknown primary guides molecularly-informed therapies despite heterogeneity. *Nat Commun*. 2022;13(1):4485. doi:10.1038/s41467-022-31866-4

7. Kleinheinz K, Bludau I, Hübschmann D, et al. *ACEseq – Allele Specific Copy Number Estimation from Whole Genome Sequencing*. Bioinformatics; 2017. doi:10.1101/210807

8. Talevich E, Shain AH, Botton T, Bastian BC. CNVkit: Genome-Wide Copy Number Detection and Visualization from Targeted DNA Sequencing. *PLoS Comput Biol*. 2016;12(4):e1004873. doi:10.1371/journal.pcbi.1004873

9. Jia P, Yang X, Guo L, et al. MSIsensor-pro: Fast, Accurate, and Matched-normal-sample-free Detection of Microsatellite Instability. *Genomics Proteomics Bioinformatics*. 2020;18(1):65-71. doi:10.1016/j.gpb.2020.02.001

10. Dobin A, Davis CA, Schlesinger F, et al. STAR: ultrafast universal RNA-seq aligner. *Bioinforma Oxf Engl*. 2013;29(1):15-21. doi:10.1093/bioinformatics/bts635

11. Uhrig S, Ellermann J, Walther T, et al. Accurate and efficient detection of gene fusions from RNA sequencing data. *Genome Res*. 2021;31(3):448-460. doi:10.1101/gr.257246.119

12. Hübschmann D, Jopp-Saile L, Andresen C, et al. Analysis of mutational signatures with yet another package for signature analysis. *Genes Chromosomes Cancer*. 2021;60(5):314-331. doi:10.1002/gcc.22918

13. Alexandrov LB, Nik-Zainal S, Wedge DC, et al. Signatures of mutational processes in human cancer. *Nature*. 2013;500(7463):415-421. doi:10.1038/nature12477

14. Rimmer A, Phan H, Mathieson I, et al. Integrating mapping-, assembly- and haplotype-based approaches for calling variants in clinical sequencing applications. *Nat Genet*. 2014;46(8):912-918. doi:10.1038/ng.3036

15. Richards S, Aziz N, Bale S, et al. Standards and guidelines for the interpretation of sequence variants: a joint consensus recommendation of the American College of Medical Genetics and Genomics and the Association for Molecular Pathology. *Genet Med Off J Am Coll Med Genet*. 2015;17(5):405-424. doi:10.1038/gim.2015.30

16. Rehm HL, Berg JS, Brooks LD, et al. ClinGen--the Clinical Genome Resource. *N Engl J Med*. 2015;372(23):2235-2242. doi:10.1056/NEJMsr1406261
