# Supplementary material for: Comprehensive genomic and transcriptomic analysis enables molecularly guided therapy options in peritoneal and pleural mesothelioma
Source: ESMO Open. 2025 Apr 1;10(4):104532. doi: 10.1016/j.esmoop.2025.104532 (PMC11999262; doi:10.1016/j.esmoop.2025.104532)
Supplement: Supplementary Figures [file mmc3.pdf]

Supplementary Figures

Supplementary Figure 1

a

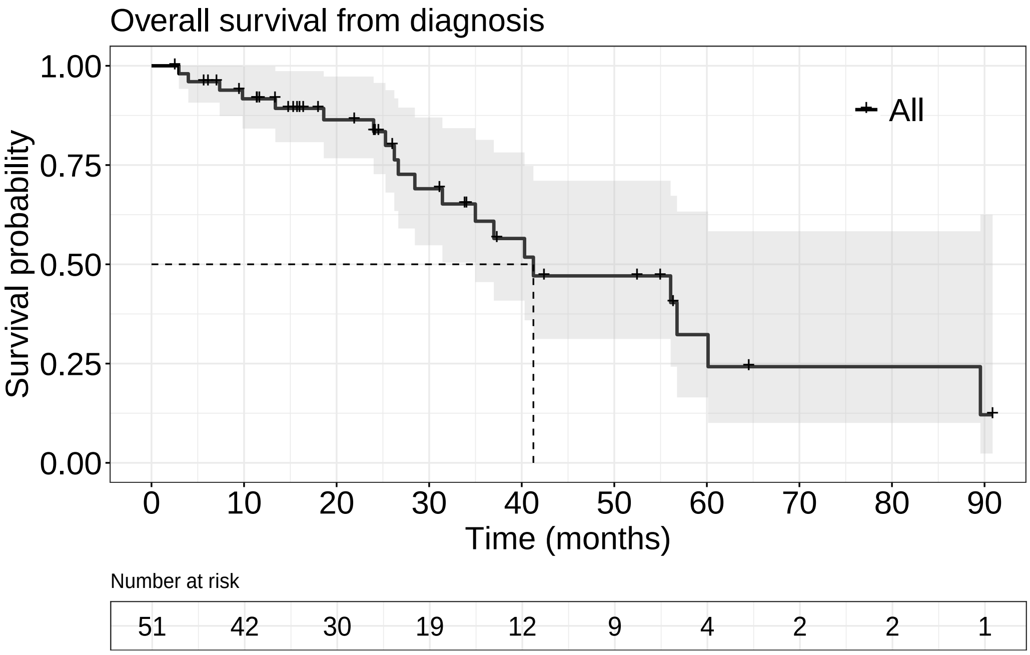

b

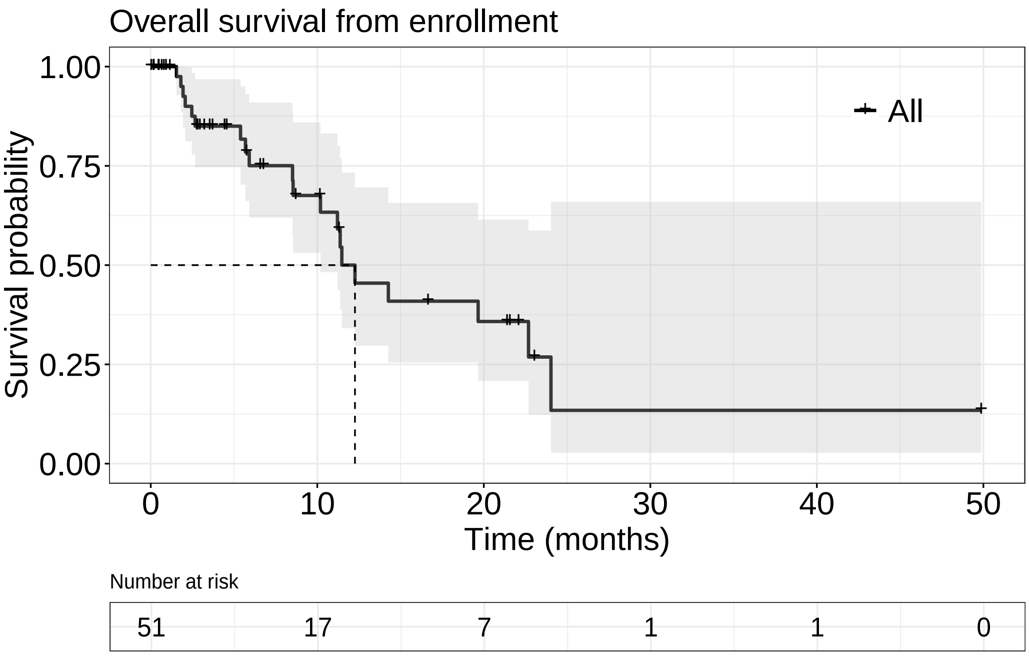

Supplementary Figure 2

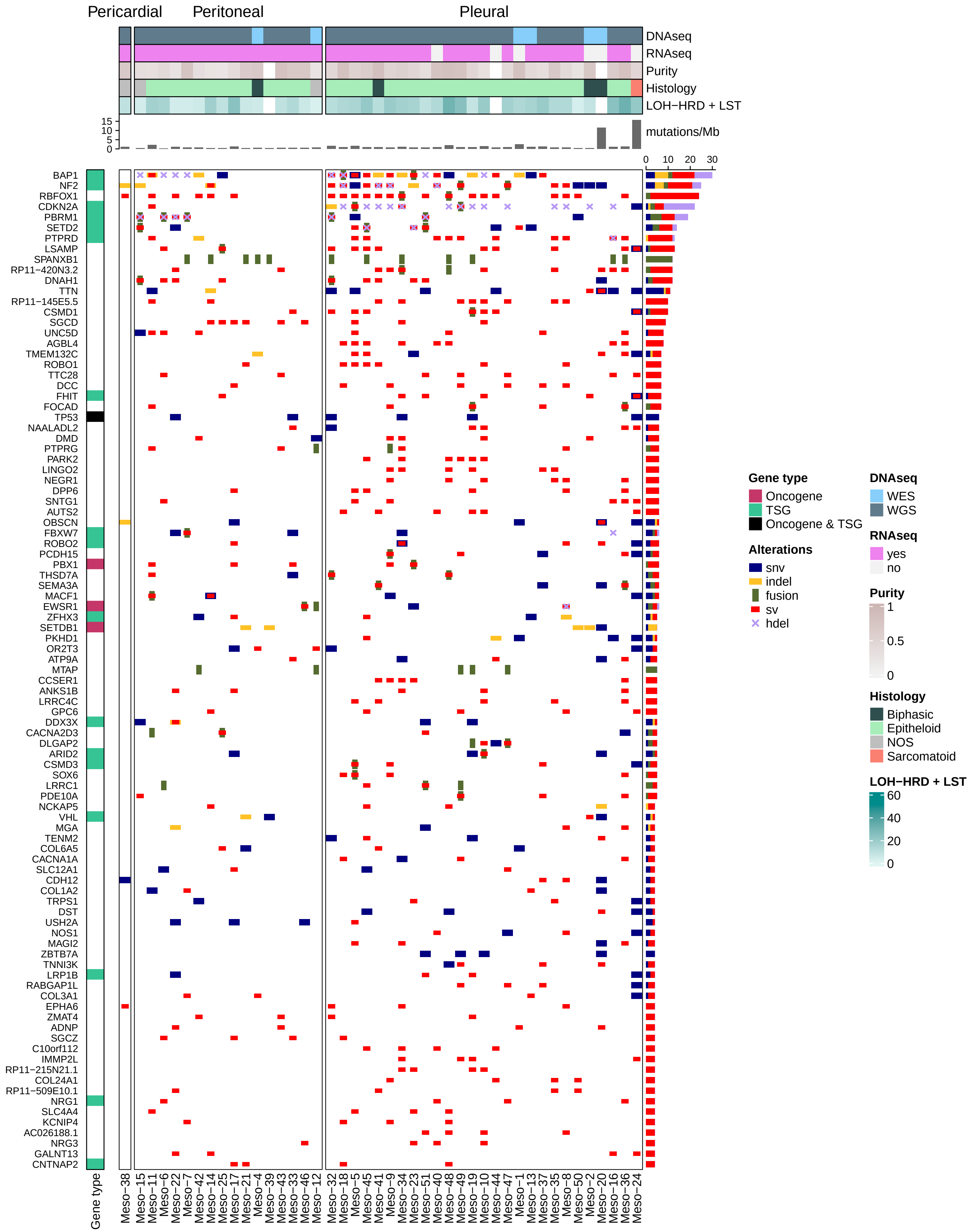

Supplementary Figure 3

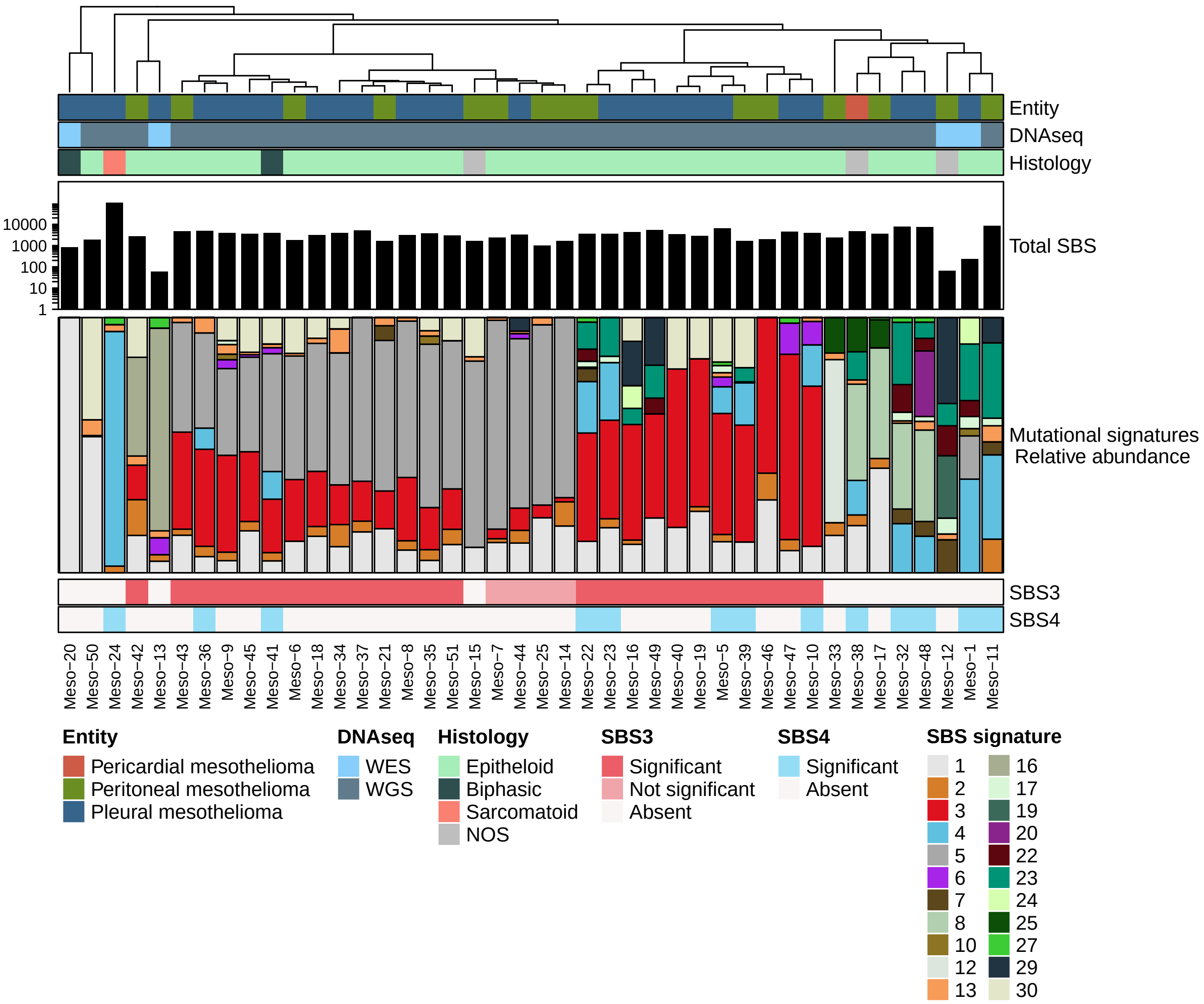

Supplementary Figure 4

a

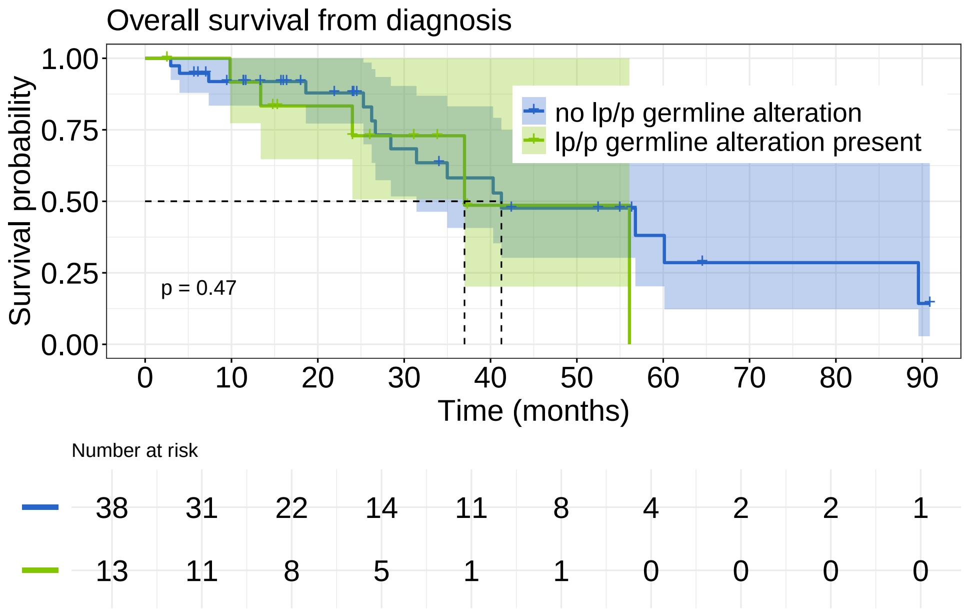

b

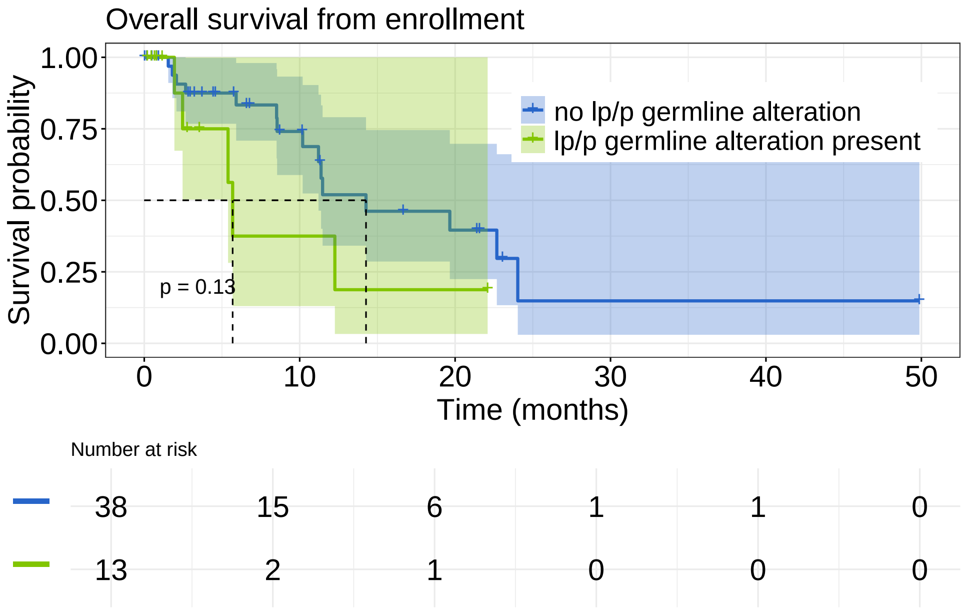

Supplementary Figure 5

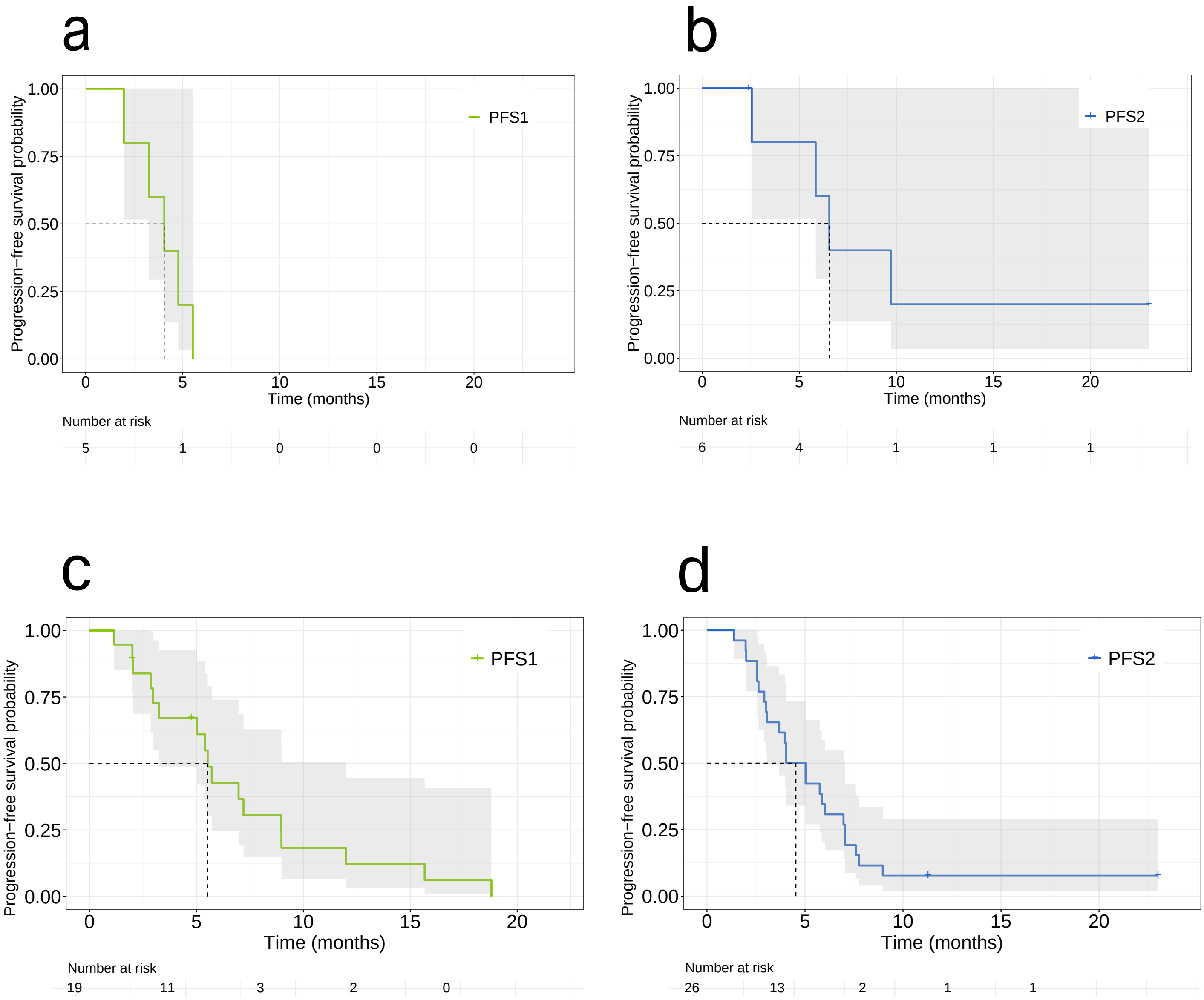

SUPPLEMENTARY FIGURE DESCRIPTIONS

Supplementary Figure 1: Kaplan-Meier plot of overall survival from A) diagnosis (median: 41.3 months) and B) enrollment (median: 12.3 months). Grey area indicates 95% confidence interval.

Supplementary Figure 2: Oncoplot including structural variants.

Supplementary Figure 3: Mutational signatures of mesothelial samples. The annotation bars at the top indicates mesothelioma entity, histology and sequencing method of the samples. The top bar plot shows the total number of SNVs on which the mutational signatures were extracted. The bottom bar plot shows the relative mutational signature exposures for each sample. The bottom annotation bars indicate whether signatures SBS 3 and 4 are detected and/or significant. Samples are hierarchically clustered by relative mutational signature exposure using complete linkage and Manhattan distance.

Supplementary Figure 4: Kaplan-Meier plot of overall survival from A) diagnosis and B) enrollment for patients with and without (likely) pathogenic germline variants.

Supplementary Figure 5: Kaplan-Meier plot displaying progression-free survival of A) the last systemic therapy before application of a therapy recommended by the molecular tumor board (PFS1, median: 4.0 months), B) the first application of a therapy recommended by the molecular tumor board (PFS2, median: 6.5 months), C) the last systemic therapy before first application of immune checkpoint inhibition (PFS1, median: 5.5 months), B) the first application of immune checkpoint inhibition (PFS2, median: 4.5 months). Grey area indicates 95% confidence interval.
