# Supplementary material for: Comprehensive genomic and transcriptomic analysis enables molecularly guided therapy options in peritoneal and pleural mesothelioma
Source: ESMO Open. 2025 Apr 1;10(4):104532. doi: 10.1016/j.esmoop.2025.104532 (PMC11999262; doi:10.1016/j.esmoop.2025.104532)
Supplement: Supplementary Material [file mmc4.docx]

**Supplementary Results**

**Recommendations of the molecular tumor board of priority one**

TBR were issued with a priority annotation. Among TBR with priority one, PARP inhibition was recommended most frequently (17/46, 37%) based on multiple molecular biomarkers such as *BAP1* alterations and/or *BAP1* underexpression (14/17), BRCAness signature AC3 (11/17), *SLFN11* overexpression (3/17) and alterations of *BRCA2* (2/17), *FANCI*, *FANCM*, *PTEN* (each 1/10). *SETD2* alterations led to recommendation of one ATR inhibitor (1/46, 2%). Tyrosine kinase inhibition was recommended in 12/46 cases (26%) based on *VEGFR/VEGFR2* overexpression (5/12), *ALK* overexpression (1/12), overexpression of *FGFR2* and *FGF1* (1/12), *NF2* alterations (3/12) and on *MET* overexpression (3/12). ICI was recommended in 6/46 cases (13%) based on *BAP1* alterations or underexpression (2/6), tumor mutational burden greater than five non-silent mutations per megabase (1/6), *NF2* alterations (1/6), *FLT1* overexpression (1/6) and overexpression of PD-L1, PD-L2 and CTLA-4 (1/6). mTOR inhibition was recommended in 4/46 cases (9%) based on somatic *VHL* alterations (2/4) and *NF2* alterations (2/4). One *NF2* alteration and one *NF1* alteration led to recommendation of two MEK inhibitors (2/46, 4%) and one *NF2* alteration led to recommendation of one YAP1 inhibitor (1/46, 2%) as part of a study opportunity. One *ERCC4* alteration led to a recommendation of platin based chemotherapy (1/46, 2%), in two cases, *MSLN* overexpression led to recommendation of a mesothelin-targeted antibody drug conjugate (2/46, 4%). One PRMT5 inhibition was recommended based on loss of *MTAP* and *CDKN2A* (1746, 2%). Recommendations with priority one fell into the following baskets: PI3K-AKT-mTOR (4/46, 9%), tyrosine kinases (11/46, 24%), RAF-MEK-ERK (2/46, 4%), DNA damage response (18/46, 39%), developmental pathways (0/46), immune evasion (6/46, 13%), cell cycle (0/46) and other (5/46, 11%).

**Additional PGV’s of note**

In a PM patient (Meso-37), a pathogenic germline variant in NF1 (NM_001042492.3:c.3739_3742del, p.(Phe1247fs)) was found with an allele frequency of 31% in control and 22% in tumor tissue. Mesothelioma is not typically associated with Neurofibromatosis type I. LOH of the wild type *NF1* allele in tumor tissue was not observed. According to our documentation, the patient showed no clinical features suggestive of Neurofibromatosis type I. Considering the patient’s age of 79 years at the time of sequencing, clonal hematopoiesis of indeterminate potential (CHiP) was discussed as the cause for the occurrence of the variant as previously described in the literature (PMID 35926323). Since somatic mosaicism could not be ruled out, the variant was reported and further clinical evaluation was recommended. The variant is not listed among the (likely) pathogenic germline variants.

PGVs in *NBN* are linked to a recessive cancer susceptibility syndrome. We identified a pathogenic germline *NBN* founder variant (NM_002485.5:c.657_661del, p.(Lys219fs)) in a PM patient (Meso-16), which had already been detected in a Dutch patient in a previous study^1^. A pan-cancer association of aforementioned variant was verified by a study using MSK-IMPACT and TCGA data, yet no association with mesothelioma could be observed^2^.

**REFERENCES**

1. Belcaid L, Bertelsen B, Wadt K, et al. New pathogenic germline variants identified in mesothelioma. *Lung Cancer Amst Neth*. 2023;179:107172. doi:10.1016/j.lungcan.2023.03.008

2. Belhadj S, Khurram A, Bandlamudi C, et al. NBN Pathogenic Germline Variants are Associated with Pan-Cancer Susceptibility and In Vitro DNA Damage Response Defects. *Clin Cancer Res Off J Am Assoc Cancer Res*. 2023;29(2):422-431. doi:10.1158/1078-0432.CCR-22-1703
